# Parental perception of facilitators and barriers to health among young children with down syndrome: a qualitative study

**DOI:** 10.3389/fped.2023.1155850

**Published:** 2023-07-11

**Authors:** Angela R. Caldwell, Yeook Kim, Nada Alshahwan, Kishore Vellody, Roxanna M. Bendixen, Kayley Renz, Tiffany Duong, Judith Dodd, Lauren Terhorst, Aviva Must

**Affiliations:** ^1^ Pediatric Health Promotion Laboratory, Department of Occupational Therapy, School of Health and Rehabilitation Sciences, University of Pittsburgh, Pittsburgh, PA, United States; ^2^ Families and Autism Research Lab, Department of Occupational Therapy, Sargent College of Health and Rehabilitation Sciences, Boston University, Boston, MA, United States; ^3^ Department of Pediatrics, School of Medicine, University of Pittsburgh, Pittsburgh, PA, United States; ^4^ Department of Rehabilitation Sciences, College of Health Professions, University of Pittsburgh, Pittsburgh, PA, United States; ^5^ Department of Sports Medicine and Nutrition, School of Health and Rehabilitation Sciences, University of Pittsburgh, Pittsburgh, PA, United States; ^6^ SHRS Data Center, School of Health and Rehabilitation Sciences, University of Pittsburgh, Pittsburgh, PA, United States; ^7^ Department of Public Health and Community Medicine, School of Medicine, Tufts University, Boston, MA, United States

**Keywords:** Down syndrome, toddler, early childhood, obesity, health, thematic analysis

## Abstract

**Background:**

Despite high rates of obesity and weight-related conditions observed in children with Down syndrome, little is known about how to prevent these conditions.

**Purpose:**

The purpose of this study was to identify parent-perceived facilitators and barriers to health for toddlers (12–36 months old) with Down syndrome.

**Materials and methods:**

We conducted in-depth, semi-structured interviews with the mothers of 25 toddlers with Down syndrome. All interviews were conducted using Zoom Video Technology, audio recorded and transcribed before being coded in NVivo software using a structured protocol. Thematic analysis was used to identify themes in perceived facilitators and barriers to health at the level of the child, family, and community. Data were triangulated using reflective journaling, video review of child meals, and member-checking techniques.

**Results:**

We identified unique themes for facilitators (on the move and sound sleep) and barriers (co-occurring conditions and eating behaviors) at the level of the child. At the level of the family and community, overarching themes that were viewed as either a facilitator or barrier, depending on the context, were identified (role models matter, time is critical, the importance of place, and social support).

**Conclusion:**

These themes can help clinicians and researchers tailor their health promotion interventions to meet the unique needs of children with Down syndrome by using strength-based approaches and providing families with the tools to overcome barriers.

## Introduction

Children with Down syndrome have an elevated risk for obesity due to altered body composition and reduced exercise capacity and energy needs (1–5). Health disparities in weight-related health outcomes during childhood include obstructive sleep apnea syndrome, gait dysfunction, non-alcoholic fatty liver disease, and adverse cardiovascular risk factor levels (1, 2, 6, 7). Despite these risks, very little information is available about preventing obesity for children with Down syndrome or other developmental disabilities (8). What is known is that individuals with Down syndrome are more likely to be physically inactive (9, 10), demonstrate poorer diet quality (11, 12), and have more sleep problems (13, 14) than their peers. Very few studies examine the health behaviors of children with Down syndrome during the first 3 years of life (15, 16). However, the preferences, habits, and behaviors established during these critical first years influence developmental trajectories and health outcomes later in life (17, 18). Because rapid weight gain has been observed in children with Down syndrome between 2 and 6 years, it is imperative that we understand how early habits are formed in this population (19). Therefore, the purpose of this study was to describe parents’ perspectives on health behaviors and the development of healthy habits in children with Down syndrome during the first 3 years of life. We were specifically interested in facilitators and barriers to health to inform the design of an intervention to reduce the risk of obesity and rapid weight gain among children with Down syndrome.

The Institute of Medicine (IOM) has prioritized helping parents promote healthy eating, increase physical activity, decrease sedentary behavior, and promote age-appropriate sleep duration during their children’s early years to prevent obesity (20). In response to the high rates of obesity in children, several obesity prevention programs have been developed to promote healthy habits during early childhood in typically developing children and children at risk due to socioeconomic factors (21–24). However, we predict that these programs will need to be modified to best meet the needs of families of young children with developmental disabilities. Children with Down syndrome face barriers that traditional obesity prevention approaches may not address. For example, because toddlers with Down syndrome commonly experience oral motor delays, swallowing issues, and other feeding problems (e.g., picky eating) (25, 26), it may be more difficult for them to meet the World Health Organization recommendation to consume 400 g, or five or more servings, of fruits and vegetables per day (27). Similarly, children with Down syndrome frequently experience sleep problems (28) and gross motor delays (29, 30) that may hinder their ability to meet recommendations related to sleep duration and engagement in physical activity early in life. There is a pressing need for interventions to help families of young children with Down syndrome build healthy routines that consider co-occurring conditions and offer creative ways to promote health regardless of developmental trajectory.

To successfully promote health and prevent obesity in children with Down syndrome, clinicians must understand the facilitators and barriers to healthy habits families experience daily. Our primary research question was: What do parents of children with Down syndrome perceive as facilitators and barriers to building healthy routines and habits? We anticipate that barriers that are unique to Down syndrome will emerge through the thematic analysis of semi-structured interviews with parents. After themes of facilitators and barriers to healthy habits are identified, they can be used to guide the tailoring of existing obesity prevention strategies. Based on the socioecological model of obesity risk for children with developmental disabilities (8), we anticipate that approaches may require modification at the level of the child, the family, and the community. The overarching goal of this study was to describe parents’ perspectives on healthy habits as they relate to their young child with Down syndrome. We used a qualitative approach to gain a deeper understanding of how parents viewed facilitators and barriers to health for their children.

## Materials and methods

### Study setting and design

We conducted a qualitative study using semi-structured interviews and thematic analysis (31, 32). All interviews were conducted remotely using Zoom technology (Zoom Video Communications, San Jose California). The university Institutional Review Board approved all research procedures, and all participants provided informed consent. This study was part of a larger mixed methods project to characterize the health behaviors of young children with Down syndrome. Recruitment and interviews occurred between June 2020 and March 2021.

### Participants

We recruited a convenience sample of 25 parents of young children with Down syndrome through social media, newspaper ads, and email blasts from specialty clinics and organizations that serve individuals with Down syndrome in the United States. To be included, parent participants had to (1) be 18 or older, (2) speak English, and (3) have a child aged 12–36 months with Down syndrome. Parents were excluded if their child used a feeding tube as their primary source of nutrition at the time of data collection. Participants were told that the interview was being conducted to help the team understand the routines and habits of families with children with Down syndrome to inform the development of a new health-promoting intervention. Interested participants completed an online survey (Qualtrics, Provo, Utah) to determine participation eligibility and provide the research team with contact information. Informed consent documents were emailed to participants before meeting with the principal investigator (via phone or video conference). After reviewing procedures and making sure all parent questions were answered, those still interested in participating were given the option to sign the informed consent document electronically and return it via email or sign a printed copy and return it in a self-addressed, stamped envelope provided by the research team. After informed consent, interviews were scheduled at times convenient for participant parents. All parent participants completed a demographic survey on REDCap and received $50 on a pre-paid university-based debit card for completing an in-depth interview.

### Semi-structured zoom interviews

Individual, in-depth interviews (35 min–60 min) were conducted via Zoom, audio-recorded using an external device, and transcribed by a qualitative data coding service at the university. The semi-structured interview script and probing questions (Table 1) were developed to align with the IOM’s obesity prevention priorities (20) and the Socioecological Model of Obesity Risk for children with developmental disabilities (8). As a form of member-checking, participant responses were paraphrased before probing questions were posed, to provide an opportunity for participants to clarify meaning and support valid interpretations. Data saturation was achieved, with no new codes or themes emerging in the data during the final rounds of thematic analysis.

**Table 1:** Semi-structured interview script.

Question	Potential probes
1. Can you describe what a typical day looks like for your child?	– *Can you tell me more about your child’s sleep behavior?*
	– *Can you tell me more about your child’s eating and nutrition?*
	– *Can you tell me more about your child’s physical activity?*
	– *Can you tell me more about your child’s use of screens?*
	– *Can you tell me more about times when your child’s movement is restricted?*
2. What kind of things influence your family’s daily routines and activities?	– *Can you tell me about things that influence what you do each day or week?*
3. Could you tell me a little bit about how COVID-19 has changed your activities and routines in each of these areas?	
4. People often think about changing things in their lives. Can you talk to me about whether or not there are things you would like to change?	– *In what ways do you think these changes would impact your family?*
5. If you were thinking about making changes, what kind of things would get in the way?	– *Do you feel you have the resources you need?*
	– *Can you tell me more about your community?*
	– *Are there other things that take up your time?*
6. What things would help you be successful at making changes?	– *Are there programs that you think might help?*
	– *How do you prefer to receive training or education?*
	– *Do you have access to resources that might help?*
7. In terms of your health, what types of behaviors or habits do you consider to be healthy ones?
	– *What does “being healthy” mean to you?*
	– *What kinds of things does a “healthy” person do?*
8. Would you describe healthy behaviors or habits differently when thinking about your child with Down syndrome?	– *Can you tell me more about why/why not?*
9. Is there anything else you would like to share with us about your family or child’s health and routines?	

The principal investigator and first author, AC, conducted all interviews. AC is a clinician-scientist and occupational therapist with 15 years of clinical expertise working with families of children with Down syndrome and interviewing families. AC acknowledges that confirmation bias based on previous experiences may have influenced the probing questions posed to families. During each interview, a second research team member was present to take field notes and offer an alternate perspective. In addition, before each interview, we sent participants a camcorder and requested that they video-record three child meals.

### Data analysis

Transcripts were uploaded into QSR International’s NVivo 12 qualitative data analysis software (Burlington, MA) for coding. The four coders had experience working with families of children with disabilities and education and training in occupational therapy. They represented diverse racial, cultural, and socioeconomic backgrounds, and each interview was coded by at least three coders using a codebook with flexibility for open coding as needed. An initial draft of the codebook was developed based on the four areas of health behaviors prioritized by the IOM (healthy eating; sleep; physical activity; and sedentary behavior). We also adapted the Socioecological Model of Obesity Risk for children with developmental disabilities (8) to include factors most relevant to children with Down syndrome. Each phrase coded was labeled as either a potential facilitator or barrier at the level of the child, family, or community.

To analyze the qualitative data, four trained coders used a coding protocol that followed the six-step process for thematic analysis (32, 33). They read each transcript to familiarize themselves with the data (step 1) prior to assigning and generating initial codes (step 2). After independently coding each interview via NVivo, coders participated in reflective journaling to take notes on key messages from each interview. Next, the team met weekly over 8 months to discuss facilitators and barriers at different levels (i.e., child, family, community) for each family and compare the findings across the families to identify potential emerging themes (step 3). The research team created a series of mind maps, visual tools to show connections or relationships of codes (34), to facilitate synthesis of codes, and to identify emerging themes. The analysis team (coders and principal investigator) met weekly to review emerging themes (step 4) and those flagged by the NVivo software system. Open codes were reviewed, and the codebook was updated iteratively over the course of data analysis to incorporate the interim findings. The analysis team held two workshops (halfway through coding and when coding was complete) to identify and define overarching themes (step 5) of facilitators and barriers before reporting the final themes (step 6). A recursive review of the initial themes was conducted in the workshops by identifying the most significant or frequently mentioned codes and combining the codes into higher-order meaningful themes. After the thematic analysis was completed, a member of our team viewed the video recordings of meals to identify child behaviors consistent with themes related to eating behaviors. Five participants representing diverse socioeconomic, racial, and ethnic backgrounds also reviewed a draft of the results of the thematic analysis, confirmed themes, and provided recommendations to improve clarity as a form of member checking.

## Results

### Participants

Of the 25 parents who participated in semi-structured interviews, all were mothers (23 biological, 2 adoptive), and most were white (*n* = 22), non-Hispanic (*n* = 20), and married at the time of the interview (*n* = 23). While diverse educational, employment, and income levels were represented (Table 2), most (*n* = 14) reported household income greater than $100,000. They ranged in age from 28 to 51 years (mean = 37). More than half of the children with Down syndrome being discussed were female (*n* = 14), and the vast majority had received early intervention services (*n* = 22). Most children had at least one sibling in the home (*n* = 21), ranging from zero to six siblings in our sample. All children with Down syndrome were between 12 and 36 months, with an average age of 25 months at the time of the interview. We received usable video-recorded child meals from 22 of 25 participants; two participants did not record child meals, and one participant’s recordings were inaccessible due to a memory card error.

**Table 2:** Participant characteristics.

Parent demographics	*N*	%
Gender		
Female (Mother)	25	100
Race		
White	23	92
Black	1	4
Asian	1	4
Ethnicity		
Non-Hispanic/Non-Latina	20	80
Hispanic/Latina	5	20
Marital status		
Married	23	92
Education level		
High school	3	12
Vocational/Associate	5	20
Bachelor’s	8	32
Graduate Degree	9	36
Employment status		
Full-time	11	44
Part-time	4	16
Stay-at-home Parent	10	40
Family income		
<$40,000	2	8
$40,000–$60,000	3	12
$60,000–$80,000	2	8
$80,000–$100,000	3	12
>$100,000	14	56
Age (years)		
Mean = 36.9; SD = 6.11; Range = 28–51		
Child demographics	*N*	%
Gender		
Female	14	56
Male	11	44
Race		
White	20	80
Black	1	4
Asian	1	4
Pacific Islander	1	4
Multi-racial	2	8
Ethnicity		
Non-Hispanic/Non-Latino	20	80
Hispanic/Latino	5	20
Number of siblings		
0	3	12
1–2	20	80
3+	2	8
Age (months)		
Mean = 25.6; SD = 7.6; Range = 12–36		

SD, Standard deviation.

### Child-level factors

We identified unique themes for facilitators and barriers to healthy habits for children with Down syndrome at the level of the child. The following themes were identified as facilitators of healthy habit formation: (1) on the move and (2) sound sleep. The barriers included: (1) co-occurring conditions and (2) eating behaviors.

### Facilitator—on the move

Parents described their toddlers with Down syndrome preferring active play and exploring their environment.

One mother whose child had recently mastered going up and down stairs explains,

“…we did go to [museum] like last month, and they have that new toddler section set up, and so it’s like stairs, and she just went up and down, and up and down, and up and down the stairs. I mean, she would’ve probably done it for a whole hour all by herself just up and down; she loved it” (P4, White mother of a 31-month-old).

This theme persisted among toddlers who were not yet walking, as described by another mother,

“And he’s very active… he’s all over… everywhere. We have baby guard—really—he’s not walking by himself yet, but he’s crawling everywhere” (P24, Asian mother of an 18-month-old).

Many parents also discussed using music and dancing to motivate physical activity. When asked about her child’s favorite activities, one mother responded,

“She loves music—any kind of music, she just starts rocking. She loves to dance.” (P25, Hispanic mother of a 23-month-old).

### Facilitator—sound sleep

Regardless of sleep apnea, a strong theme of sound sleep emerged: parents described their toddlers with Down syndrome as good sleepers. A mother of three explained,

 “She sleeps great… she’s my only child who has slept great” (P18, White mother of an 18-month-old). Similarly, another mother of three children shared, “…he sleeps through the night… of all my kids, he taught himself to fall asleep on his own” (P17, White mother of a 27-month-old).

In addition to sleeping through the night, mothers reported their children fell asleep quickly, with one sharing,

“she does really good about staying in bed at nighttime. And she usually falls asleep within five minutes” (P7, Hispanic mother of a 29-month-old).

### Barrier—co-occurring conditions

Parents’ most frequent barriers to healthy habits were related to co-occurring medical conditions. Despite reports of sound sleeping, parents described medical concerns related to sleep apnea. One mother explained,

 “…the doctor told me that she [has] progressed to like severe sleep apnea. So, she’s sleeping; at some point, she’s not able to breathe. She sleeps well, but I don’t think she’s getting any quality sleep because of the sleep apnea because she’s tossing and turning a lot. But other than that, she’s a good sleeper compared to my other kids” (P8, Black mother of a 36-month-old).

Other co-occurring conditions parents discussed as barriers to the development of healthy habits included dysphagia, reflux, and gastrointestinal issues. One mother shared,

“she has dysphagia…she’s had multiple swallow studies, and she’s still aspirating quite a bit… she’s on thickeners, so she cannot take anything just straight liquid she also has some GI issues, some constipation, she has really bad reflux, so she [takes] medicine in her bottle” (P15, Hispanic mother of a 15-month-old).

Another mother described fears related to repeated choking associated with dysphagia,

“she is more prone to choking…I don’t feel like her skills are quite there yet. And we’ve had a couple of choking incidents, and it’s not any fun to have to give your child back blows to get that thing out of their throat, so we proceed with caution” (P7, Hispanic mother of a 29-month-old).

Episodes of gagging and choking were confirmed upon review of video-recorded child mealtimes submitted by parents.

### Barrier—eating behaviors

While parents often described their children as “good eaters,” they also described eating behaviors that served as barriers to creating healthy habits. Parents describe their children with Down syndrome as lacking appropriate satiety signals and tending to eat whatever amount of food they are offered. One mother explained this,

“…[she] loves to eat… She doesn’t really have a… I guess I would say like a full sensor that we’re aware of, she will just keep asking to *eat and eat and eat*” (P7, Hispanic mother of a 29-month-old). Another mother shared a similar experience, “he’ll never tell us to stop. Like he’ll just *eat and eat and eat*. …we have to read a lot from his cues, too. Like we can tell, he’s done when he starts to play around. Or when he just starts to get really slow” (P9, White mother of a 27-month-old).

One mother described feeling like she needed to stop her child from eating,

“when she starts eating, I feel like she keeps eating. And there are times where she’ll tell us that she’s all done, but I feel like a lot of times she might just keep eating, and we have to cut her off” (P22, White mother of a 19-month-old).

Parents of toddlers with Down syndrome also described strong food preferences and picky eating behaviors. One mother explained,

“all she wants is carbs and sugar. So like, if we just gave her 500 mini muffins, that’s all she would eat. But we try to make her eat other things” (P25, Hispanic mother of a 23-month-old).

Another mother expressed frustration relying on formula for nutrition,

“she is such a picky eater… usually, her lunch consists of SpaghettiOs or Chef Boyardee, which we’re trying to expand off of, because I don’t want her to just be eating that. …a lot of the nutrients [are] coming from this formula, so we kind of really rely on that” (P3, White mother of a 30-month-old).

One mother described increased pickiness over time,

 “So, she used to eat all sorts of things and has since become very picky. She loves carbs, of course…and fruits. Has trouble with meats and vegetables” (P14, White mother of an 18-month-old).

Video review of child mealtimes confirmed the tendency for children in this sample to eat carbohydrates and starches first during meals.

### Family-level factors

Two major themes emerged as factors influencing the development of healthy habits among children with Down syndrome early in life: (1) role models matter, and (2) time is critical. Interestingly, these themes were described in competing ways as both facilitators and barriers depending on the family context.

### Role models matter

Parents, siblings, and extended family members were all described as role models that could positively or negatively influence child health behaviors. We have highlighted examples of this theme related to nutrition, physical activity, and the use of screens.

Regarding nutrition, siblings were described as powerful role models for mealtime behaviors. One mother who described her child’s restricted eating behaviors also provided a strategy she used to increase dietary variety,

“she’s a major imitator… she never liked strawberries. So… I’ll have her sister eat—start eating it, and after she sees her eating, and then she’ll start saying, “eat, eat,” that means she wants some” (P8, Black mother of a 36-month-old).

On the other hand, not all parents described positive role models for nutrition. A mother who described deep ties to her community explained how the foods valued in her culture could serve as a barrier to healthy habits,

“Mexican families especially that love refried beans, they love all the good delicious things that are maybe not so great to eat all the time… On both sides of our family, there’s, you know, health issues, things related to weight… that run in our families: heart disease, things like that, you know?” (P15, Hispanic mother of a 15-month-old).

Many parents expressed a desire to be positive role models in terms of physical activity. One mother explained,

“I want us…to be examples for her so that she can see, ‘Mom and dad go for walks, let me do the same thing… my sister is playing a sport… let me, you know, find my thing so I can do it too” (P15, Hispanic mother of a 15-month-old). Another parent described how her family had prioritized physical activity, “…my husband and I are very into being active, exercising…so we…one thing we’ve really tried to do just even with the kids as they’re little, just go outside at least once a day and do something: walk, you know, ride bikes, something like that” (P14, White mother of an 18-month-old).

Alternatively, another mother admitted,

 “I’m addicted to my phone. She’s seen me on it, and so she discovered, ‘Oh, Mom’s got [her phone] in her pocket.’ …that whole day was terrible, ‘cause then all she wanted to do was grab our phones” (P4, White mother of a 31-month-old).

### Time is critical

Families also described how work setting (home vs. office) and other competing demands influenced the time available to build healthy family routines, both as a facilitator and a barrier.

A mother who worked full-time described commute time as a barrier,

“Traffic really affects us in our house because we both commute to work, and so if there’s a wreck on the interstate, that could really cause problems getting home at night” (P7, Hispanic mother of a 29-month-old).

Whereas another described the flexibility of working in the home and a short commute as facilitators,

“I’m a stay-at-home mom, so my schedule is very flexible. My husband right now— ‘cause we live on base, so he doesn’t have much of a commute; he can come home for lunch, that helps a lot” (P4, White mother of a 31-month-old).

Many parents reflected on a recent transition to working from home due to the COVID-19 pandemic. One mother shared,

“I’m mostly working from home, so I have—you know, my husband and I have more time to have things ready… and she’s not like overly hungry waiting for dinner at this point… the kids are sitting down eating at the same time” (P14, White mother of an 18-month-old).

Parents of young children with Down syndrome also described balancing several weekly appointments and other scheduling conflicts as competing demands for time, thus serving as a barrier to healthy habit development. One mother explained how this takes away from other responsibilities,

“Sometimes things like how busy I am on my day off, if [child] has a lot of…like if she has her therapies, and then if she has other afternoon appointments, sometimes I don’t have time to do all the things that I would like to do on my day off, like go grocery shopping, clean the house, that sort of thing” (P7, Hispanic mother of a 29-month old). Another mother shared similar experiences, “…our schedule is insane. Let’s see. My daughter has activities—my other two kids go to school. Some days are in person; some days are remote. So, I work in the schools here, so I’m gone every day. My husband’s here working from home. The other two are coming and going depending on the day. In the evenings, my daughter has dance three nights a week… It’s just crazy. I’m still in college. When I’m at home, it’s just crazy” (P25, Hispanic mother of a 23-month-old).

### Community-level factors

Regarding community factors, two themes emerged that could serve as either a facilitator or a barrier depending on family context: (1) the importance of place and (2) social support.

### Importance of place

Parents of young children with Down syndrome acknowledged the critical role of their place (proximity to resources and the state policies related to health services) as factors that influence their ability to support their child’s health.

Families described the types of community in which they resided (e.g., urban, rural) as a potential facilitator or barrier. One mother describes the benefits of living in a walkable, suburban community,

“the daycare is right up the street, so… we always try to stay outside, and you know, stay active, and like walk to the playground or the park, or ride her little bike around. So, we do have access to those sorts of things” (P14, White mother of an 18-month-old). Another shared similar benefits, “Our community has a lot of really great walking trails. They have a pool. They have playgrounds” (P15, Hispanic mother of a 15-month-old).

One mother described barriers experienced living in a rural community,

“We have parks, but unfortunately, this area is very rural, so like there’s no… group meetings. And before I had [child] I was an OB nurse, and nobody ever talked about Down syndrome… there are a few people in the area that are older that have Down syndrome, but… they were in group homes, like you never saw them in the community” (P13, White mother of a 26-month-old).

Parents also discussed the proximity of specialists and access to care based on state policies as influencing the health of their toddler with Down syndrome. A mother who had recently moved described variations between states,

“[former state] is our only place that we’ve experienced special needs support, and they were outstanding. So going from there to here has been somewhat of a disappointment. They have resources, but they’re harder to come by. And speech therapists, they’re very hard to come by around here” (P2, White mother of a 31-month-old).

Another mother described barriers to accessing virtual services across state lines,

“normally, you’d see the whole pediatric team, and they’d evaluate like, you know, physical therapy, speech, occupational therapy, but because we’re out of state, like we—we could only meet with [the physician]” (P17, White mother of a 16-month-old).

Many parents described feeling satisfied with their services, expressing their areas had access to “so many resources,” or that their providers were “super, super, super knowledgeable.”

### Social support

Parent participants also cited the presence of social support as a facilitator and a lack of social support as a barrier to healthy habit formation. A mother of seven children describes how her son’s weekly appointments have added complexity to their schedule,

“…as a homeschooling family, it’s been hard… ideally, we’ve been trying to find… a co-op or something one day week, [so] I had a day to focus on him and his doctor’s appointments and therapies” (P19, White mother of a 19-month-old).

Many parents described a desire to connect with other families. One mother explains,

“I would love… to have like playdates for him, whether with, you know, [other children with] Down syndrome or, you know, the same type of playgroups I did for my daughters” (P17, White mother of a 16-month-old).

Others described the importance of having the support of grandparents to help them promote healthy habits. One mother explained,

“they were seeing their grandparents pretty regularly, and some of their aunts and uncles, and… I feel like that has helped a lot with development, and stress, and just, you know, trying to stay healthy and have a good attitude about things” (P4, White mother of a 31-month-old).

Themes at the level of the family and the community represented a spectrum of experiences perceived as either facilitators or barriers based on contextual factors. It is important to acknowledge that at the level of the child, not all participants reported the same barriers. For example, not all parents said their toddlers experienced feeding problems. Similarly, a few parents reported that their toddler did not sleep soundly and frequently woke at night. Each parent participant described unique and unifying experiences that influenced the health behaviors of toddlers with Down syndrome. We have focused on overarching themes (Table 3), but varying experiences highlight the ongoing need for family-centered strategies that are tailored to each child and family.

**Table 3:** Facilitators (+) and barriers (−) by socioecological level.

Themes	Sub-themes	Type	Exemplar codes	Definitions
Child-level				
On the move	Prefers active play	+	Activity level	Description of the child’s activity level
	Loves to dance	+	Dance	Child preference for music and dancing
Sound sleep	Sleeps through night	+	Sleep quality	The onset of sleep or ability to stay asleep
	Falls asleep quickly	+	Sleep quantity	The duration of sleep (how long)
Co-occurring conditions	Risk of sleep apnea	−	Obstructive sleep apnea	A condition characterized by repeatedly interrupted breathing during sleep (symptoms include loud snoring, pauses in breathing, restless sleep)
	Swallowing issues	−	Pharyngeal functions	Swallowing function of a child; factors that impact efficient swallowing and airway protection (e.g., aspiration, choking, cough)
Eating behaviors	Lack of satiety	−	Control of eating	Whether the child can voluntarily stop eating when they feel full or not
	Prefers carbohydrates	−	Preferred foods	The childs preferred foods
	Restricted diet	−	Food refusal, picky eating	Eating a limited variety of foods or unwillingness to try new foods, despite the ability to eat a broader diet
Family-level				
Role-models matter	Siblings as motivators	+	Sibling effect	The impact of the sibling on the child with Down syndrome
	Parents as models	+	Family physical activity	Family activity routine, such as walking or going to a park
	Traditional foods	−	Family screen use	Description of family screen use influencing screen time of the child with Down syndrome
		−	Family meals	Types of foods that family commonly eats at home, whether they eat together or not
Time is critical	Flexible work setting	+	Family dynamics	Distinct factors of family function/roles that influence child health (e.g., work situation, marital status, travel)
	Competing demands	−	Competing time demands	Limited time to build healthy habits for child with Down syndrome (e.g., work responsibilities, other children, other appointments)
Community-level				
Importance of place	Proximity to resources	+/−	Access to inclusive facilities	Access to places to engage in physical and recreational activity for children with varying abilities.
	Access to services	+/−	Access to specialists	If family can access inclusive providers, early intervention services, medical specialists
	Financial strain	−	Cost	How much resources cost is described (e.g., services, food, opportunities for activity)
Social support	Presence or absence of social support	+/−	Community and others	Local community factors that influence parent and child health (e.g., peers, family, friends, respite providers)

## Discussion

Using semi-structured in-depth interviews with parents and thematic analysis guided by a socioecological framework, we aimed to identify facilitators and barriers to building healthy habits and routines for young children with Down syndrome. In the category of child factors, a preference for active play (*on the move*) and a tendency to sleep through the night (*sound sleep*) emerged as facilitators that helped parents promote healthy routines for their toddlers with Down syndrome. *Co-occurring conditions* (e.g., sleep apnea, dysphagia, heart conditions) and dysfunctional *eating behaviors* (e.g., lack of satiety signals, restricted diet, picky eating, preference for starches) emerged as barriers that impeded parents’ ability to build healthy routines for their toddlers with Down syndrome.

At the levels of the family and community, overarching themes emerged that were discussed as either facilitators or barriers based on each family’s unique context and perspective. Specifically, the themes of *role models matter* and *time is critical* emerged as central to promoting healthy behaviors at the family level. Community themes revolved around access, with the importance of place and social support being highlighted by the parents in our sample. Ease of access to parks, walking trails, specialists, and opportunities to promote health was valued. The level of social support from friends, family, and other parents of young children with Down syndrome also emerged as a key contributor to building healthy habits. Parents lacking access and social support viewed these as community-level barriers, whereas those with access and support reported these as community facilitators.

The findings in this study support existing research that has identified co-occurring conditions, family role models, access to inclusive services, and time (i.e., competing demands) as factors that influence the health behaviors of individuals with Down syndrome (35–37). Social support has been linked to health-related quality of life and parenting stress among caregivers of children with Down syndrome (38–40). Therefore, it is not surprising that social support emerged as an important theme, but our study suggests parents perceive social support as being a critical component to health promotion for the entire family, including their child with Down syndrome.

Eating patterns and feeding problems have been linked to nutrition-related problems, such as obesity, among individuals with Down syndrome (1, 25, 26). The parents in our sample raised several concerns about eating behaviors that they viewed as barriers to healthy habit formation. Notably, many parents described their child as unable to regulate their own food intake; several reporting that their child would continue to eat preferred foods until the food was gone or taken away. Increased levels of leptin have been observed in children and youth with Down syndrome, potentially influencing leptin resistance and an ability to suppress appetite and regulate body weight (41). Our findings suggest that toddlers with Down syndrome may already be experiencing problems regulating appetite and satiety. This idea aligns with emerging evidence that children at increased risk for obesity may be less able to regulate caloric intake than their peers (42–44).

It is widely established that individuals with Down syndrome do not meet recommendations for physical activity and engaged in less physical activity than peers throughout childhood (10, 45). Furthermore, the duration and intensity of physical activity declines as children with Down syndrome get older (10, 45). Nevertheless, high levels of physical activity emerged as a perceived facilitator to healthy habit development for young children with Down syndrome in our thematic analysis. Our finding is consistent with other qualitative studies that found that children with Down syndrome prefer active play during early childhood. However, these same reports indicate that this preference for active play shifts to a preference for sedentary tasks during middle childhood (46, 47). Future research is needed to explore why children with Down syndrome appear to shift from more active to more passive activities as they develop. Nonetheless, innovative interventions are needed to support ongoing interest, motivation, and opportunities for children with Down syndrome to engage in physical activity.

It was surprising that parents in our sample overwhelmingly described their toddlers with Down syndrome as good sleepers. Children with Down syndrome are prone to sleep issues, including high rates of obstructive sleep apnea (48), lower sleep efficiency (49), higher incidences of night awakenings, and higher duration of wake after sleep onset (50). Many parents reported a history, current diagnosis, or increased risk for obstructive sleep apnea, despite good sleeping behaviors. This aligns with prior research that found parent-reported child sleep problems are not predictive of the severity or presence of sleep apnea (51, 52). Others acknowledged that sleep quality may be impacted by obstructive sleep apnea and other medical issues. That sleep emerged as a facilitator of healthy habits is noteworthy, as the disconnect between sleep health and parent-reported sleep behavior may indicate that there are fewer behavioral manifestations of sleep problems among toddlers with Down syndrome. Additional research is needed to understand the relationships between sleep behaviors and sleep outcomes (e.g., duration, night waking, sleep onset) among toddlers with Down syndrome.

Our findings should be viewed in the context of some study limitations and strengths. We acknowledge that the biases and experiences of the analysis team, each of whom has experience providing therapy to young children with developmental disabilities, can impact several aspects of study implementation and analysis. It is a limitation that the former experiences of the study team likely influenced interview questions, coding, and the identification of themes. We attempted to minimize bias by developing an interview guide, informing participants of our expertise and motivation, and following a detailed coding protocol. Another limitation of our study is that, like most qualitative studies, we report qualitative data on a convenience sample of participants; inferences to the greater population are not supported. Furthermore, our sample of families was largely recruited through specialty clinics at the beginning of the Covid-19 pandemic. This is a limitation, as those who volunteered to participate during this unusual time may be inherently different from other parents of young children with Down syndrome. Finally, our findings are further limited by the lack of diversity of our sample with respect to parent gender, race, and marital status. Therefore, our analyses may not represent the experiences of fathers and non-white or non-married mothers. Future work is warranted to understand the lived experiences of families of young children of Down syndrome within minoritized communities. Study strengths include: (1) diversity of backgrounds and perspectives of members of the research team, (2) use of member checking and video recordings to confirm the thematic analysis, and (3) a rigorous protocol for coding and thematic analysis that included weekly meetings and workshops.

In summary, we have described parent perceptions of facilitators and barriers to promoting healthy habits and routines for young children with Down syndrome. Parents described child sleep and preference for active play as facilitators and co-occurring medical conditions and eating behaviors as barriers at the level of the child. Positive role models and time to build routines emerged as facilitators, whereas negative role models and lack of time emerged as barriers at the level of the family. Finally, access to resources and social support emerged as facilitators, with lack of resources and lack of support representing barriers, at the level of the community. Additional research is needed to understand the underlying mechanisms of the child behaviors reported by parents. Understanding the factors that cause or contribute to dysregulated hunger and satiety signals, declining levels of physical activity over time, and sleep problems among children with Down syndrome are necessary to prevent obesity and weight-related health issues. Based on these themes, it is clear that existing obesity prevention interventions will require modification for children with Down syndrome to address child-level feeding issues and integrate tailored strategies to promote healthy habits for children with co-occurring conditions, such as obstructive sleep apnea. Also evident is the need for community-level interventions to improve inclusivity by increasing access to resources, building accessible spaces for physical activity, and educating leaders on how to include children with Down syndrome and their families in community activities. Future research is needed to adapt existing obesity prevention interventions and to develop toolkits and other appropriately tailored supports to meet the needs of families of young children with Down syndrome and other developmental disabilities.

## Data Availability

The datasets presented in this article are not readily available because the data collected that informed this study are qualitative in nature, inclusive of videos and interview transcripts. To protect the privacy of the participants, we do not have truly anonymous data to share. Requests to access the datasets should be directed to Angela Caldwell (ARL78@pitt.edu).
